# SARS-CoV-2 infects neurons, astrocytes, choroid plexus epithelial cells and pericytes of the human central nervous system in vitro

**DOI:** 10.1099/jgv.0.002009

**Published:** 2024-07-12

**Authors:** Ruth Haverty, Janet McCormack, Christopher Evans, Kevin Purves, Sophie O'Reilly, Virginie Gautier, Keith Rochfort, Aurelie Fabre, Nicola F. Fletcher

**Affiliations:** 1 Veterinary Sciences Centre, University College Dublin, Belfield, Dublin 4, Ireland; 2 Research Pathology Core Facility, Conway Institute of Biomedical and Biomolecular Research, University College Dublin, Belfield, Dublin 4, Ireland; 3 Centre for Experimental Pathogen Host Research, School of Medicine, University College Dublin, Belfield, Dublin 4, Ireland; 4 Conway Institute of Biomedical and Biomolecular Research, University College Dublin, Belfield, Dublin 4, Ireland; 5 School of Biotechnology, Dublin City University, Glasnevin, Dublin 9, Ireland; 6 Department of Histopathology, St. Vincent’s University Hospital, Dublin 4, Ireland

**Keywords:** brain, COVID-19, neurological, SARS-CoV-2, tropism

## Abstract

Severe acute respiratory syndrome coronavirus-2 (SARS-CoV-2) infection is associated with neurological sequelae including haemorrhage, thrombosis and ischaemic necrosis and encephalitis. However, the mechanism by which this occurs is unclear. Neurological disease associated with COVID-19 has been proposed to occur following direct infection of the central nervous system and/or indirectly by local or systemic immune activation. We evaluated the expression of angiotensin-converting enzyme-2 and transmembrane protease, serine 2 (TMPRSS2) in brain tissue from five healthy human donors and observed low-level expression of these proteins in cells morphologically consistent with astrocytes, neurons and choroidal ependymal cells within the frontal cortex and medulla oblongata. Primary human astrocytes, neurons, choroid plexus epithelial cells and pericytes supported productive SARS-CoV-2 infection with ancestral, Alpha, Delta and Omicron variants. Infected cells supported the full viral life cycle, releasing infectious virus particles. In contrast, primary brain microvascular endothelial cells and microglia were refractory to SARS-CoV-2 infection. These data support a model whereby SARS-CoV-2 can infect human brain cells, and the mechanism of viral entry warrants further investigation.

## Introduction

Severe acute respiratory syndrome coronavirus-2 (SARS-CoV-2), a member of the Coronaviridae, is a positive-sense, single-stranded RNA virus with a genome size of ~30 kb [1]. Responsible for the COVID-19 pandemic, it has caused approximately 7 million confirmed deaths [2]. While infection is primarily associated with respiratory disease [1], chronic complications are increasingly recognized in recovered patients [3,4]. These include neurological sequelae, including fatigue and cognitive dysfunction and insomnia, as well as gastrointestinal symptoms, arthralgia and other chronic symptoms, collectively referred to as post-acute sequelae of SARS-CoV-2 infection [4]. In addition, neurological symptoms, including loss of smell and taste, are observed in acutely infected individuals [4]. The pathogenesis of SARS-CoV-2 neurological disease is incompletely understood and may be due to viral tropism for cells of the central and peripheral nervous systems and/or as a result of systemic inflammation or other mechanisms [4].

A number of studies have investigated whether SARS-CoV-2 can directly infect the central nervous system (CNS). Studies have reported viral antigen in choroid plexus organoids with disruption of the blood-cerebrospinal fluid (CSF) barrier [3,5] and productive viral infection of neural progenitor cells and brain organoids [6]. In contrast, Pedrosa *et al*. did not detect SARS-CoV-2 infection of human neurospheres [7]. *In vivo*, SARS-CoV-2-like particles have been detected in brain microvascular endothelium [8]. A further study investigating SARS-CoV-2 infection of neural organoids together with mouse and human brain detected viral antigen in neuronal cells and cortical neurons in post-mortem human brain tissue [9]. Therefore, while there is increasing evidence that SARS-CoV-2 is neurotropic, there remain questions on the main cell types within the CNS that support viral infection, the role of angiotensin-converting enzyme-2 (ACE2) and other receptors in viral infection and the ability of infected cells to support the full viral life cycle remain unclear.

SARS-CoV-2 infection may also cause neurological disease independent of direct infection of the CNS, through immune activation and cytokine production. A study of COVID-19 patients with neurological disease observed elevated proinflammatory cytokines, including IL-6, IL-8 and IL-18 in serum andCSF [10], while another study reported elevated IL-6, IL-8 and TNF-α in CSF [11]. The disruption of blood-brain barrier (BBB) integrity and the activation of immune-competent cells within the CNS following peripheral immune activation have been reported, with microglial activation and lymphocyte infiltration [12]. However, the exact role of immune activation and direct viral infection of the brain in the pathogenesis of SARS-CoV-2 neurological disease remains unclear.

To investigate whether SARS-CoV-2 can infect cells of the human CNS, we investigated the expression of the SARS-CoV-2 entry receptors ACE2 and TMPRSS2 in post-mortem human brain tissue, which are required for infection of other cell types including airway epithelium [13,14]. We then evaluated a panel of primary cells isolated from human brain tissue, including primary brain microvascular endothelial cells (BMECs), astrocytes, neurons, pericytes, choroid plexus epithelial cells/choroidal ependymal cells and microglia, for their ability to support SARS-CoV-2 infection. Finally, the ability of permissive cells to support infection with Alpha, Delta and Omicron BA.1 variants was investigated.

## Methods

### Immunohistochemistry to determine ACE2 and TMPRSS2 expression in normal human brain tissue

Normal human brain tissue was obtained from the MRC Edinburgh Brain and Tissue Bank (MRC Database Number BBN). Sequential 4-µm sections of formalin-fixed, paraffin-embedded normal brain tissue from five donors, or human colon or prostate as positive control tissues for ACE2 and TMPRSS2, respectively, were dewaxed and rehydrated, and antigen retrievalantigen retrieval was performed in a PTLink with EDTAEDTA buffer and citrate buffer and stained on an AS Link 48 autostainer. After incubation with primary antibodies [0.8 µg ml^−1^ (TMPRSS2 ab109131, Abcam), 1 µg ml^−1^ (ACE2 ab15348, Abcam) or a universal negative control reagent containing mouse, rabbit and goat IgG (Enzo Life Science, NY, USA)], sections were incubated with an HRP polymer, revelated using diaminobenzidine chromogen and counterstained with haematoxylin (EnVision Flex HRP High pH Kit, K8002, Agilent Technologies). The stained slides were scanned at 20× magnification using the Aperio AT2 digital slide scanner (Leica Microsystems). Slides were evaluated for positive TMPRSS2 and ACE2 staining and likely cells expressing each receptor by a board-certified pathologist (AF).

### Cell lines, primary cells and antibodies

VeroE6 cells (ATCC CRL-1586) were propagated in Dulbecco’s Modified Eagle’s Medium (DMEM) + GlutaMAX-I (Gibco) supplemented with 10 % FBS and 1 % non-essential amino acids (Gibco). Commercially available primary human neurons (HNs; 1520–10), primary human astrocytes (HAs; 1800-10), primary human choroid plexus epithelial cells (HCPEpiCs; 1310) and primary human brain vascular pericytes (HBVPs; 1200) were obtained from ScienCell (Carlsbad, CA, USA). Primary cells were cultured as indicated by ScienCell and maintained in neuronal medium supplemented with 1 % neuronal growth supplement (HNs), astrocyte medium supplemented with 1 % astrocyte growth supplement (HAs), epithelial cell medium supplemented with 1 % epithelial cell growth supplement (HCPEpiCs) or pericyte medium supplemented with 1 % pericyte growth supplement (HBVPs), together with 2 % FBS (ScienCell) and 1 % penicillin/streptomycin (ScienCell). Primary human BMECs (ACBRI 376) were obtained from Cell Systems (Kirkland, WA, USA) and maintained in serum-containing basal medium (Cell Systems) supplemented with 1 % CultureBoost (Cell Systems) and 0.05 % Bac-Off (Cell Systems). Immortalized human microglia (P10354-IM) were obtained from Innoprot (Bizkaia, Spain) and cultured in microglial basal medium (Innoprot) supplemented with 1 % microglial growth supplement (Innoprot), 5 % FBS and 1 % penicillin/streptomycin. Primary cells from at least two donors for each cell type were evaluated. All cells were maintained at 37 °C, 5 % CO_2_.

Primary antibodies used for immunofluorescent staining and neutralization assays were as follows: SARS-CoV-2 Spike mAb anti-rabbit IgG (Sino Biological, Beijing, China), dsRNA K1 mAb anti-mouse IgG2a (Scicons, Susteren, The Netherlands) and recombinant anti-ACE2-neutralizing mAb anti-mouse IgG1 (Sino Biological, Beijing, China). Fluorescent secondary antibodies Alexa Fluor 488 and 594 goat anti-rabbit and anti-mouse, respectively, were obtained from Invitrogen.

### Virus culture and isolates

SARS-CoV-2 (2019-nCoV/Italy-INMI1) obtained from the European Virus Archive Global (GenBank accession MT077125.1) was propagated in VeroE6 cells. All virus used in this study was at passage 3. Cells were inoculated with a multiplicity of infection (MOI) 0.01 for 2 h and washed with PBS, and the medium was replaced with DMEM containing 2 % FBS. When cultures were fully infected (exhibited greater than 90 % cytopathic effect), flasks were freeze thawed three times and supernatants collected and clarified at 3500 r.p.m. for 30 min at 4 °C. Supernatant was collected, aliquoted and stored at −80 °C. TCID_50_ (50 % tissue culture infectious dose) was performed in VeroE6 cells in quadruplicate and infectious titre determined using the Reed-Muench method^20^. Clinical isolates representing SARS-CoV-2 variants including Alpha (CEPHR_IE_B.1.1.7_0221, GenBank accession ON350867, passage 2), Delta (CEPHR_IE_AY.50_0721, GenBank accession ON350967) and Omicron (CEPHR_IE_BA.1_0212, GenBank accession ON350968, passage 2) were isolated from SARS-CoV-2-positive nasopharyngeal swabs from the All-Ireland Infectious Disease cohort [13]. These were isolated and amplified on VeroE6/TMPRSS2 cells (#100978), obtained from the Centre for AIDS Reagents at the National Institute for Biological Standards and Control [15].

### SARS-CoV-2 infection and neutralization

Cells were plated on collagen/poly-l-lysine-coated 24-well plates and, 24 h later, inoculated with SARS-CoV-2 for 1 h at 37 °C, washed and incubated at 37 °C. At the indicated times post-infection, cells were fixed for 1 h with 4 % paraformaldehyde (PFA) for immunofluorescent staining. Alternatively, to evaluate infectious viral titre within cells or released into the culture medium, supernatant was harvested and an equal quantity of culture medium added to cells which were freeze thawed three times and centrifuged at 3000 r.p.m., and the supernatant was collected from cellular lysate. Cellular supernatant (released virus) or lysate (intracellular virus) was titrated on VeroE6 cells. Seventy-two hours post-titration, TCID_50_ was calculated according to the method of Reed and Muench [16]. Eight replicates per experiment were used to calculate two TCID_50_ values from quadruplicate technical replicates. Infection was quantified at 72 h post-infection based on optimization of SARS-CoV-2 infection levels in VeroE6 cells as a positive control. To ensure that input virus was not present in supernatants, PBS from the final wash 1 h post-infection was titrated on VeroE6 cells to confirm that no infectious virus was present. To allow comparisons between viral isolates and cell types, for experiments in which SARS-CoV-2 Alpha, Delta and Omicron infectivity was evaluated, immunopositive cells were enumerated and then normalized to astrocytes (the cell type that had the highest density 24 h post-seeding when cells were infected) and the Italy_INMI1 isolate which had the highest titre. This allowed comparisons of relative infectivity between cell types and viral variants.

To evaluate the ability of neutralizing anti-ACE2 antibody to inhibit infection of human neural cells, 10 µg ml^−1^ antibody was incubated with the cells for 1 h at 37 °C prior to inoculation with SARS-CoV-2.

### Cell proliferation assay

To assess cell viability prior to SARS-CoV-2 inoculation, a 3-(4,5-dimethylthiazol-2-yl)-2,5-diphenyl-2H-tetrazolium bromide (MTT) assay (Vybrant MTT Cell Proliferation Assay; Thermo Fisher Scientific) was performed on VeroE6 and primary human brain cells 24 h post-plating as described in SARS-CoV-2 infection and neutralization section, to mimic the timepoint at which cells were inoculated with SARS-CoV-2. Cells were treated with 0.05 % Triton X-100 (TX-100) for 5 min as a positive control for cellular toxicity. The MTT assay was performed according to the manufacturer’s instructions and as previously described [17].

### Immunofluorescent staining and microscopy

Target cells (1×10^5^ ml^−1^) were plated on bovine type 1 collagen (BMEC), poly-l-lysine-coated Thermanox coverslips (Nunc Thermo Fisher, Roskilde, Denmark) or 24 well cell culture plates and incubated for 24 h. Cells were exposed to SARS-CoV-2 as described in SARS-CoV-2 infection and neutralization section and fixed with 4 % PFA for 1 h at 37 °C. Primary antibodies anti-SARS-CoV-2 spike (Sino Biological, 20 µg ml^−1^), K1 anti-dsRNA (Scicons, 2 µg ml^−1^) or IgG isotype matched control antibody (Enzo Life Sciences, USA) were incubated for 1 h at room temperature and washed, and secondary antibodies Alexa 488/Alexa 594 (Invitrogen, 2 µg ml^−1^) were incubated for 1 h at room temperature. Cells were mounted with ProLong Gold containing DAPI counterstain and imaged using an Olympus FV3000 confocal microscope or enumerated by manual counting using a Zeiss Axio Imager epifluorescent microscope. Confocal data were collected as z-stacks and deconvolved, and maximum intensity was projected. Infectivity is expressed as focus-forming units per millilitre (FFU/ml) of virus.

### Real-time quantitative reverse-transcription polymerase chain reaction (RT-qPCR)

RT-qPCR assays were performed on an ABI 7500 Fast Real-Time PCR machine (Applied Biosystems, Waltham, MA, USA) in a total volume of 20 µl, containing 5 µl of template and TaqMan Fast Virus 1-Step MasterMix (Applied Biosystems). Primer sequences and concentrations and thermal cycling conditions for SARS-CoV-2 nucleocapsid gene were as previously described [18]: forward primer, GACCCCAAAATCAGCGAAT; reverse primer, TCTGGTACTGCCAGTTGAATCTG; and probe, FAM-ACCCCGCATTACGTTTGGTGGACC-IBFQ. Primers and probes were all used at 500 nM. Cycling conditions were reverse transcription (RT)(50 °C – 600 s), 95 °C – 30 s, 45 cycles (95 °C – 5 s, 60 °C – 30 s).

### Statistical analysis

Statistical analyses were performed using Student’s t-test or one-way ANOVA in Prism 9.1.2 (GraphPad), with *P* < 0.05 being considered statistically significant and corrected for multiple comparisons when required (Bonferroni).

## Results

### Astrocytes, neurons and choroidal ependymal cells from the human CNS express ACE2 and TMPRSS2

**Fig. 1.**
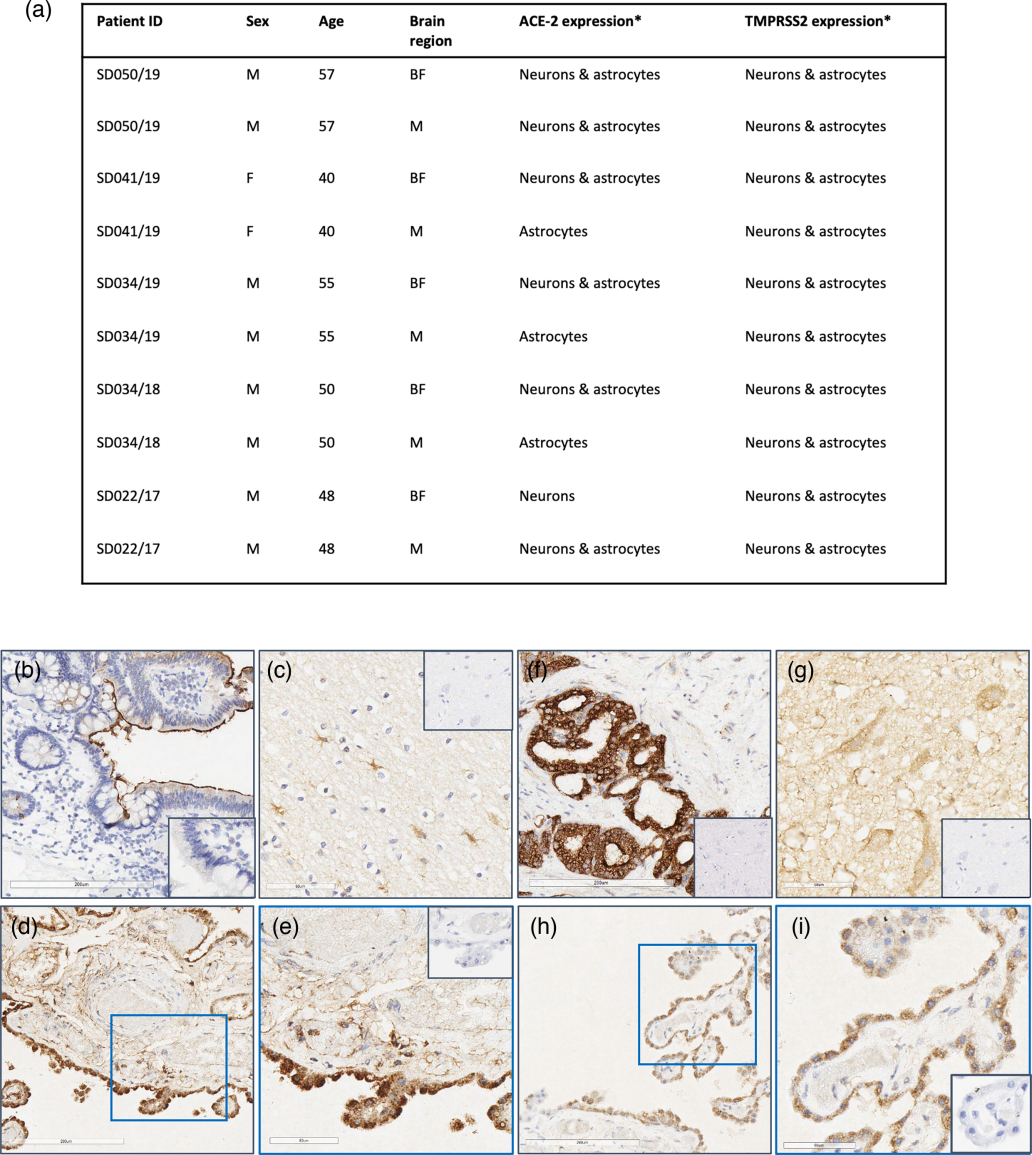
ACE-2 and TMPRSS2 expression in normal human brain tissue. (**a**) Sections of formalin-fixed, paraffin-embedded frontal cortex and medulla from five healthy donors were stained for immunoreactivity to ACE-2 and TMPRSS2. Low-level ACE-2 and TMPRSS2 expression was observed in cells morphologically consistent with astrocytes and neurons within both brain regions. Brain regions: BF, medulla oblongata; M, anterior frontal convexity. (**b**) ACE2 immunohistochemical (IHC) staining in human colon (positive control). Inset: IgG control. (**c**) ACE2 expression in frontal cortex. Inset: IgG isotype control. (**d**) ACE2 staining in choroid plexus. (**e**) Magnified area highlighted in the blue box in (d). Inset: IgG isotype control. (**f**) TMPRSS2 IHC staining in human prostate (positive control). Inset: IgG isotype control. (**g**) TMPRSS2 staining in human frontal cortex. Inset: IgG isotype control. (**h**) TMPRSS2 IHC staining in choroid plexus. (**i**) Magnified area highlighted in the blue box in (h). Inset: IgG isotype control. Bar: 200 µm (**b, d, f, h**). Bar: 60 µm (**c, e, g, i**). *Based on morphological assessment of cell type.

Frontal cortex and medulla oblongata from five healthy human donors were evaluated for expression of the SARS-CoV-2 entry receptor, ACE2, together with TMPRSS2, which is reported to prime the spike protein of SARS-CoV-2 and is required for viral entry to nasopharyngeal epithelium (Hoffmann *et al*., 2020)[19]. Low-level cytoplasmic expression of both receptors was observed in cells morphologically consistent with neurons and astrocytes in both brain areas examined from all five donors (Fig. 1a). In a single donor for which choroid plexus was available, choroidal ependymal cells were immunoreactive to both ACE2 and TMPRSS2 (Fig. 1b). The expression of both receptors was lower in brain tissue compared with positive control tissue (human colon-ACE2; prostate-TMPRSS2)(Fig. 1b). No non-specific staining was observed in IgG controls (Fig. 1b; insets and Fig. S1, available in the online version of this article).

### SARS-CoV-2 infects HNs, astrocytes, pericytes and choroid plexus epithelial cells

**Fig. 2.**
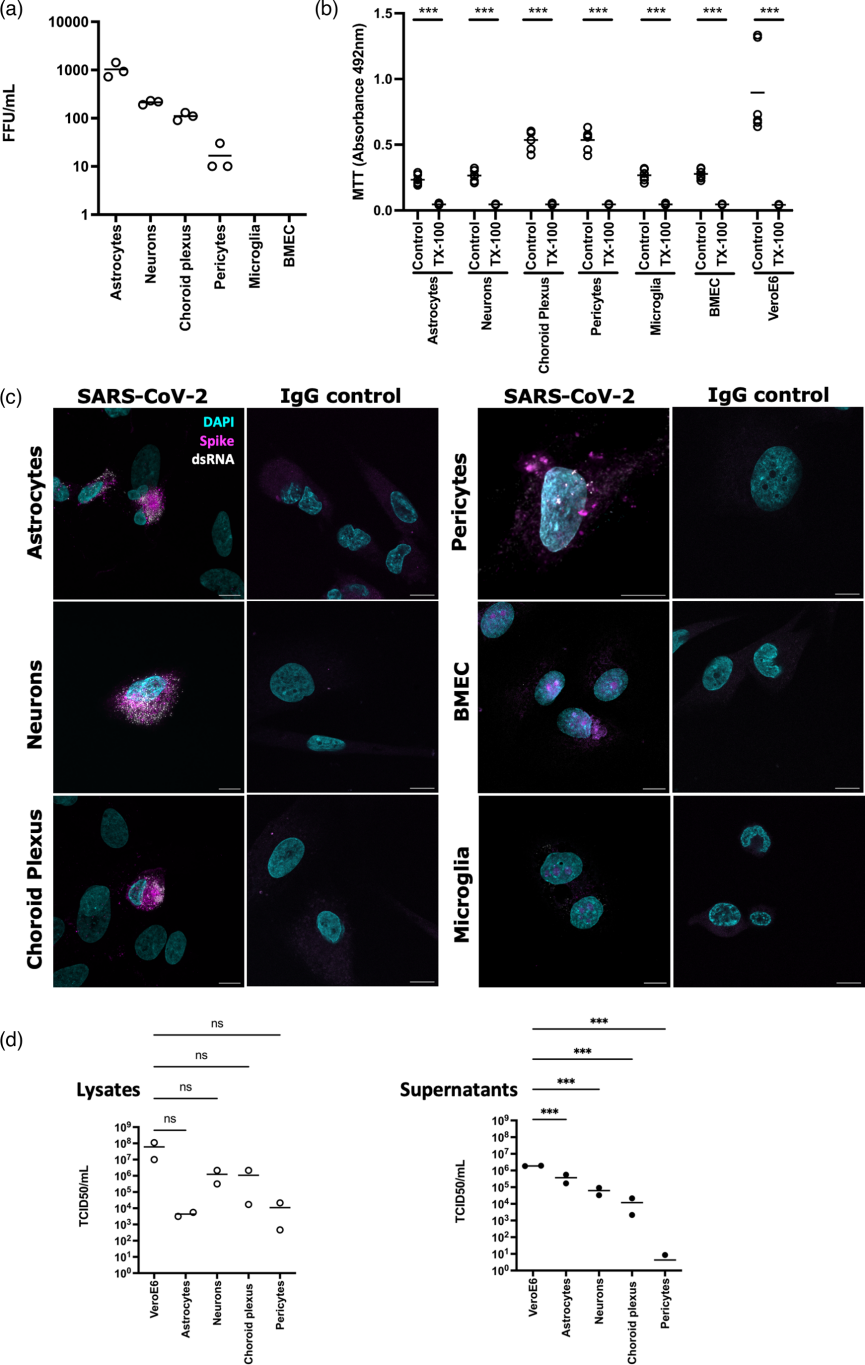
SARS-CoV-2 infects HNs, astrocytes, pericytes and choroid plexus epithelial cells. (**a**) Primary HNs, astrocytes, choroid plexus epithelial cells, brain vascular pericytes, microglia and BMECs, were exposed to SARS-CoV-2 at an MOI of 0.1 for 1 h. After 72 h, cells were fixed and immunolabelled for anti-SARS-CoV-2 spike protein and dsRNA, and infected cells were enumerated. Data are presented as FFU/ml. (**b**) Primary human brain cells, or VeroE6 cells, were cultured for 24 h and maintained in culture medium or treated with 0.05 % TX-100 for 5 min as a positive control for cellular toxicity. MTT was assayed by quantification of absorbance at 492 nm. (**c**) Representative images of SARS-CoV-2-inoculated cells. Anti-SARS-CoV-2 spike protein is shown in magenta, dsRNA (Scicons K1 mAb) in white and DAPI in blue. Images were taken at 10x or 20x depending on the size of the imaged cells. Scale bars: 10 µm. (**d**) To quantify viral loads within and released from infected brain cells, cellular lysates (left panel) and supernatants (right panel), respectively, from primary human brain cells and VeroE6 cells were titrated on VeroE6 cells and TCID_50_ quantified after 72 h. A one-way ANOVA was performed to determine statistical differences between viral loads in brain cells compared with VeroE6 cells. ***P < 0.001, **P < 0.01, *P < 0.05. ns, not significant. *N* = 3 independent experiments.

Primary HNs, astrocytes, choroid plexus epithelial cells, BMECs, brain vascular pericytes and immortalized human microglia were inoculated with SARS-CoV-2 (Italy-INMI1 at an MOI of 0.01). Neurons, astrocytes and choroid plexus epithelial cells supported SARS-CoV-2 infection, with low levels of infection observed in pericytes. Infected cells were enumerated, and, due to the lack of cytopathic effect in any brain cell type following SARS-CoV-2 infection, infectivity was calculated as FFU/ml (Fig. 2a). Astrocytes supported the highest levels of infection at 72 h post-infection, with approximately 25-fold higher levels of infected cells than that of pericytes. Similar numbers of infected cells were observed at 48 h post-infection (data not shown). Prior to infection, cells were confirmed to be viable and proliferating using an MTT assay (Fig. 2b). Anti-SARS-CoV-2 spike and dsRNA staining was observed in infected cells (Fig. 2c). In contrast, no infection of BMEC or microglia was observed (Fig. 2a and c).

### SARS-CoV-2-infected HNs, astrocytes and choroid plexus epithelial cells support the full virus life cycle

To establish whether SARS-CoV-2-permissive brain cells were capable of supporting the full virus life cycle, virus released by infected cells into the extracellular medium, and intracellular virus in lysed cells, was quantified by titration on VeroE6 cells 72 h post-infection of primary human brain cells. Primary HNs, astrocytes, choroid plexus epithelial cells and pericytes supported the full virus life cycle, containing infectious virus within cells (lysate) and released from infected cells (supernatants) when titrated on VeroE6 cells and quantified using TCID_50_ assays (Fig. 2d). While no significant difference was observed in intracellular viral loads between cell types, brain cells released significantly less virus into the extracellular medium than VeroE6 cells (Fig. 2d). Neurons and choroid plexus epithelial cells displayed similar levels of intracellular infectious virus and virus released into the extracellular medium. In contrast, astrocytes released more infectious virus into the extracellular medium, with a titre of 3.675×10^5^ ml^−1^ compared to 4.39×10^3^ ml^−1^ for cell lysate (Fig. 2d). Pericytes were permissive to infection but released low levels of virus, with a 3-log decrease in virus titre in the supernatant compared to intracellular virus (Fig. 2d). Infectious virus was not detected in the supernatants of cells following PBS washes post-infection, confirming that virus detected in supernatants or cell lysates was not residual input virus (data not shown). Furthermore, infectious virus was not detected from either cellular lysate or supernatant of BMEC or microglia.

### SARS-CoV-2 infection of human brain cells is inhibited by an anti-ACE2 antibody

**Fig. 3.**
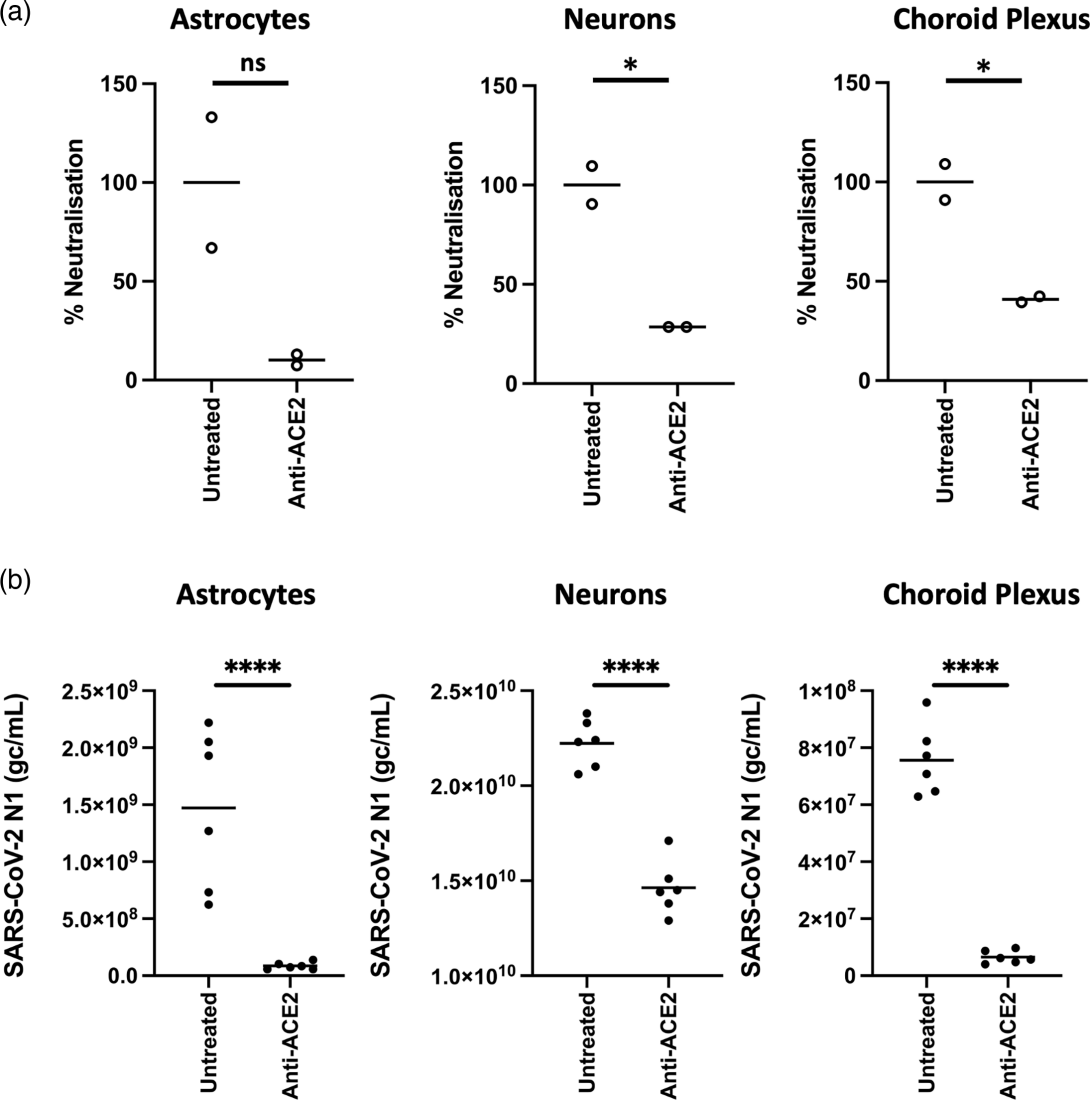
Primary HAs, neurons and choroid plexus epithelial cells were incubated with ACE2-neutralizing antibody for 1 h (10 µg ml^−1^) before being inoculated with SARS-CoV-2 (Italy_INMI1) at an MOI of 0.1 for 1 h. (**a**)Seventy-two hours post-infection, cells were fixed and immunolabelled for anti-SARS-CoV-2 spike protein, and infected cells were enumerated. Data are presented as % neutralization relative to the untreated control. (**b**) Cells were lysed and SARS-CoV-2 nucleocapsid (N1) gene was quantified by RT-qPCR. Data are presented as SARS-CoV-2 N1 genome copies per millilitre. ****P < 0.0001, **P < 0.01, *P < 0.05. ns, not significant. *N* = 3 independent experiments.

Given the low levels of ACE2 in human brain tissue, we next determined whether infection of primary human brain cells was inhibited by a neutralizing ACE2 antibody. In neurons and choroid plexus epithelial cells, a significant decrease in infection in the presence of anti-ACE2 antibody was observed compared to control infected cells. While a decrease in the number of infected astrocytes was observed, this decrease was not statistically significant (Fig. 3a). Due to the extremely low levels of SARS-CoV-2 infection in pericytes, it was not possible to evaluate anti-ACE2 neutralization in these cells. Using RT-qPCR to quantify SARS-CoV-2 N1 gene, a significant decrease in levels of N1 RNA in all three cell types following treatment with anti-ACE2 neutralizing antibodies was observed (*P* < 0.0001)(Fig. 3b).

### SARS-CoV-2 variants infect human brain cells

**Fig. 4.**
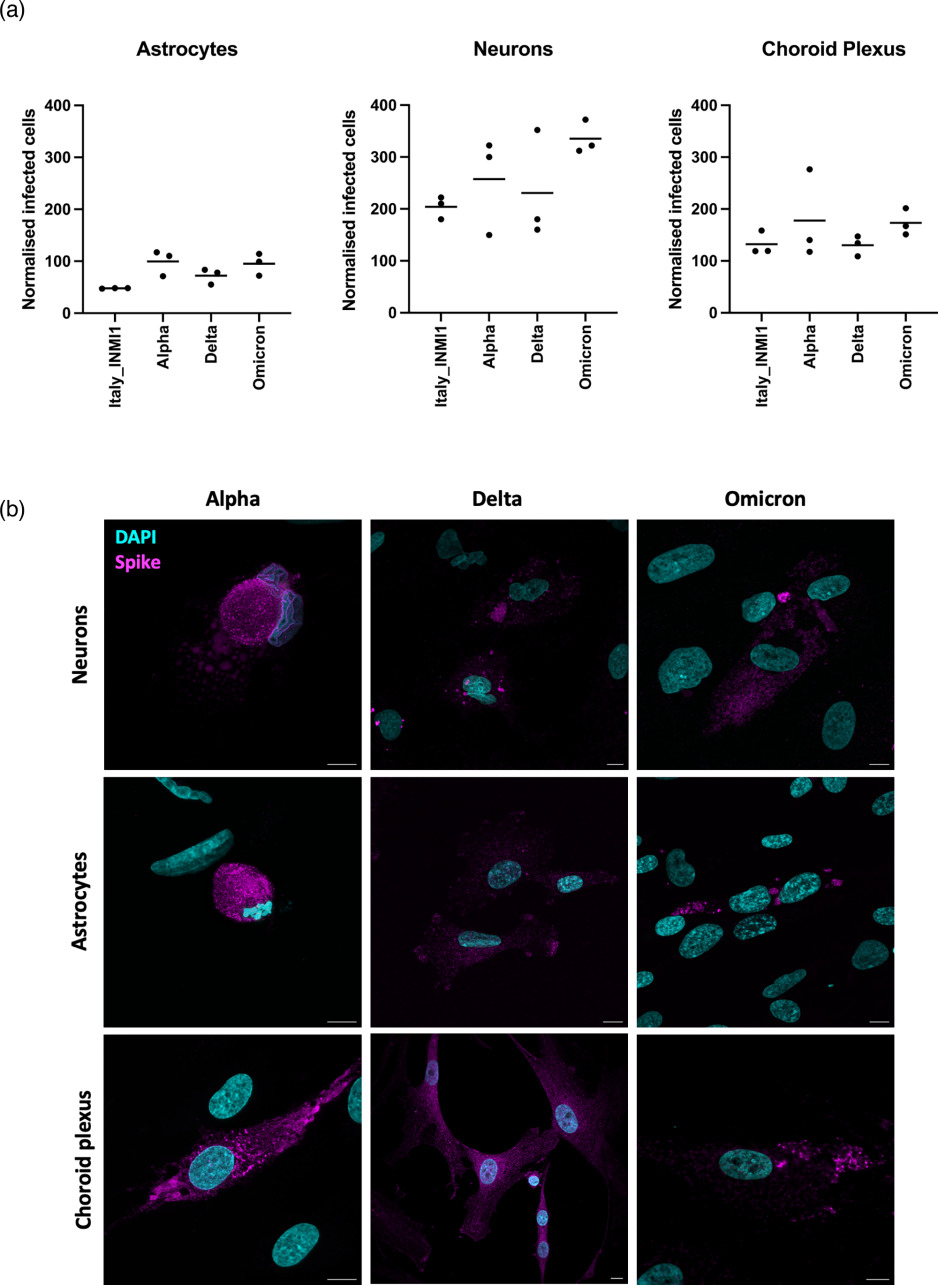
SARS-CoV-2 variants infect primary human brain cells. Primary HNs, astrocytes and choroid plexus epithelial cells were inoculated with SARS-CoV-2 variants (Italy_INMI1, Alpha, Delta and Omicron) at an MOI of 0.1 for 1 h. After 72 h, cells were fixed and immunolabelled for anti-SARS-CoV-2 spike protein, and infected cells were enumerated. Data are presented as infected cell counts adjusted for virus titre and cell number. (**b**) Representative confocal images of SARS-CoV-2-infected cells. Anti-SARS-CoV-2 spike protein is shown in magenta and DAPI in cyan. Scale bars: 10 µm.

Cells that were permissive to SARS-CoV-2 infection (neurons, astrocytes and choroid plexus epithelial cells) were infected with three clinical isolates of SARS-CoV-2 variants representing Alpha, Delta and Omicron BA.1. Primary HNs, astrocytes and choroid plexus epithelial cells supported infection by all three variants. Similar to our observations with Italy-INMI-1, BMEC and microglia did not support infection with SARS-CoV-2 variants. Pericytes were not infected with these variants, likely due to the lower titre of the clinical isolates compared with SARS-CoV-2 Italy-INMI-1 and therefore the lower likelihood of infection due to low permissiveness of these cells to infection (data not shown). Neurons supported the highest levels of infection by all variants, and astrocytes and choroid plexus epithelial cells were infected at similar levels (Fig. 4a). Representative images of SARS-CoV-2-infected cells revealed SARS-CoV-2 anti-spike immunoreactivity in infected cells (Fig. 4b).

## Discussion

There is a growing body of evidence of neurological manifestations of COVID-19, and therefore, understanding the mechanisms of SARS-CoV-2 neuropathology will have a significant impact on treatment strategies. Neurological sequelae in COVID-19 patients are diverse and include headache, encephalitis, vasculopathy and vasculitis including stroke, haemorrhage and cerebral thrombosis [20]. The underlying mechanism by which SARS-CoV-2, directly or indirectly, causes these manifestations is currently unclear, and there may be multifactorial mechanisms. Moreover, peripheral nervous system sequelae including Gullain-Barre syndrome, peripheral neuropathy and myopathy, likely due to severe pulmonary disease, have been reported [20,21]. Recently, medium- and long-term follow-up studies revealed an increased risk of dementia, migraine, Alzheimer’s disease and other cognitive defects 12–24 months post-infection. However, it is unclear whether SARS-CoV-2 exacerbated previously undiagnosed neurological disease was associated with the Delta variant or confined to older age groups [22,23].

SARS-CoV-2 binds to target cells in the nasopharyngeal epithelium and respiratory tract at the initial stages of infection via the cellular receptor ACE2. In these tissues, SARS-CoV-2 binding to ACE2 is followed by viral spike S’ subunit proteolytic cleavage at the cell surface by the transmembrane serine protease-2 [14]. However, it is currently unclear whether cells of the CNS express these receptors or whether the mechanism of viral entry to CNS cells is the same as in other cell types such as respiratory epithelium. In the present study, cells morphologically consistent with astrocytes, neurons and choroidal ependymal cells within normal human brain tissue expressed low levels of these SARS-CoV-2 entry factors, and receptor distribution was mainly cytoplasmic which has previously been reported [24]. Similar profiles of receptor mRNA and antigen expression were observed in human brainstem in previous studies [25,26]. However, we were unable to demonstrate ACE2 or TMPRSS2 protein expression in primary brain cells cultured *in vitro* using immunofluorescence (data not shown). A limitation of this study is that receptor expression was not evaluated by Western blot, and it is possible that *in vitro* culture of primary cells used in this study may have altered or downregulated receptor expression. Recent studies have highlighted that alternate receptors may be used for SARS-CoV-2 entry to the cells of the CNS, with neuropilin-1, CD147 and DPP4 reported as potential SARS-CoV-2 entry factors for astrocytes [27,28], and that TMPRSS2 is not required for infection of neurons [29]. While we observed partial neutralization of SARS-CoV-2 infection using a neutralizing anti-ACE2 antibody, we were unable to fully inhibit infection indicating that the mechanism of viral entry to the cells of the CNS may involve a different pathway to that reported for nasopharyngeal epithelium and other sites of infection [14]. Furthermore, it is possible that clathrin-mediated endocytosis of ACE2, rather than blocking-related reduction of viral binding, could have accounted for the reduction in infection, similar to the reported internalization mechanism for SARS-CoV-2 spike protein [30]. Therefore, future studies investigating the mechanism of SARS-CoV-2 entry to the cells of the CNS are warranted.

A recent, large-scale imaging study of CNS pathological changes in COVID-19 patients revealed a loss of grey matter in several cortical areas including the limbic areas linked to the olfactory and gustatory system, providing a potential mechanism for the loss of taste and smell experienced by some individuals during acute infection [31]. Moreover, a post-mortem study investigating SARS-CoV-2 antigen expression in the CNS of COVID-19 patients identified viral antigen in olfactory mucosa, in particular olfactory epithelial cells and neurons, which support a model by which SARS-CoV-2 may invade the brain via the olfactory mucosa and the olfactory tract of the CNS through the cribriform plate [32]. Studies in non-human primates reported SARS-CoV-2 infection of neurons in the olfactory circuit of macaques, with a robust inflammatory response, blood vessel disruption and a downregulation of ACE2 expression [33] and rare SARS-CoV-2 infection of brain endothelial cells, with neuroinflammation, microhaemorrhage, neuronal degeneration and hypoxic-ischaemic injury similar to autopsy studies in humans [34]. The present study revealed that primary HNs, astrocytes, pericytes and choroid plexus epithelial cells are permissive to SARS-CoV-2 infection and support the full virus life cycle *in vitro*. Several studies have used a range of neuronal cell lines, organoids and induced pluripotent stem cells (iPSC) to investigate the tropism of SARS-CoV-2 for the CNS and have confirmed viral infection of these models, with disagreement as to which cell types support infection. Torices *et al.* [35] demonstrated that cells of the neurovascular unit, astrocytes and microglia had the highest expression of ACE2 and TMPRSS2 and demonstrated SARS-CoV-2 infection of the human neuroblastoma cell line, SH-SY5Y. In contrast, Pellegrini *et al.* [5] demonstrated that SARS-CoV-2 does not infect neuronal cells within brain organoids, but instead cells of the choroid plexus epithelium, while Ramani *et al.* [36] demonstrated preferential infection of neurons within brain organoids. Using iPSC-derived neurons and astrocytes, Kettunen *et al.* observed that SARS-CoV-2 infected neurons at a low level, but not astrocytes [29]. Few studies have investigated SARS-CoV-2 infection of primary, differentiated human cells. In agreement with the current study, Proust *et al.* [37] demonstrated SARS-CoV-2 infection of primary human pericytes and astrocytes, with cell death in pericytes, which was not observed in the present study. While few studies have reported the presence of viral RNA in brain tissue from patients with COVID-19, a recent study demonstrated viral RNA and SARS-CoV-2 spike expression in five patients with histological changes suggestive of SARS-CoV-2 neuropathology. The main cell type expressing viral antigen was astrocytes and neurons, in agreement with the present study [37].

Various mechanisms of neuroinvasion by SARS-CoV-2 have been proposed, including viral disruption and invasion across the BBB and blood-CSF barrier, via cranial nerves, including the olfactory nerve or trigeminal nerve, or anterograde or retrograde axonal transport (reviewed by Bauer *et al*. [38]). The role of these potential mechanisms in SARS-CoV-2 neuroinvasion is currently unclear and warrants further investigation. The central role of astrocytes in maintaining BBB integrity is of potential significance given our observation, in agreement with others, that astrocytes are potentially a significant target of SARS-CoV-2 replication. Disruption of astrocyte homeostasis could lead to BBB breakdown and viral and inflammatory mediator entry to the CNS, particularly if other mechanisms of viral entry play a role, such as viral invasion via cranial nerves. Similarly, choroid plexus epithelial cells form tight junctions and maintain blood-CSF barrier function, so disruption of this barrier opens a potential further portal of viral entry to the brain. Many therapeutics, such as those used as human immunodeficiency virus antivirals, do not readily cross the BBB and reach therapeutic concentrations within the CNS [39]. Furthermore, several neurotropic viruses can cause neurotransmitter disturbances and biochemical alterations in neurons [40]. SARS-CoV-2 has been reported to affect dopaminergic (DA) signalling. Yang *et al.* [41] demonstrated that iPSC-derived DA neurons senesced in response to SARS-CoV-2 infection and viral N gene and DA neuron marker tyrosine hydroxylase colocalized in neurons of the substantia nigra autopsy samples of COVID-19 patients. The degeneration of DA neurons in the substantia nigra has been associated with the development of Parkinson’s disease (PD). Clinically, it has been observed that PD patients suffer from increased COVID-19-associated mortality compared to non-PD patients [42]. Therefore, further studies investigating the ability of SARS-CoV-2 to replicate in the brain *in vivo* and the consequences of direct and indirect effects of the virus on brain homeostasis are warranted. Moreover, therapeutic strategies should consider the ability of antivirals to penetrate the brain in therapeutic concentrations.

In conclusion, we report that cells morphologically consistent with astrocytes, neurons and choroid plexus epithelial cells express ACE2 and TMPRSS2 in the human CNS, but these receptor levels are low compared with other tissues, including prostate, colon and respiratory tract, that express high receptor levels. Using primary human brain cells cultured *in vitro*, we confirmed that astrocytes, neurons, choroid plexus epithelial cells and pericytes support the full virus life cycle and release infectious virions. In addition to ancestral SARS-CoV-2, clinical isolates of Alpha, Delta and Omicron infected astrocytes, neurons and choroid plexus epithelial cells. This data supports a model whereby SARS-CoV-2 is neurotropic, and further studies investigating the *in vivo* relevance of these data, and the mechanism of entry of SARS-CoV-2 to these cells, are warranted.

## supplementary material

10.1099/jgv.0.002009 Uncited Fig. S1.

## Abbreviations

ACE2: angiotensin-converting enzyme-2
BBB: blood-brain barrier
CNS: central nervous system
CSF: cerebrospinal fluid
DMEM: Dulbecco’s Modified Eagle’s Medium
FFU: focus-forming unit
HA: human astrocytes
HBVP: human brain vascular pericytes
HIV: human immunodeficiency virus
IHC: immunohistochemical
iPSC: induced pluripotent stem cells
MOI: multiplicity of infection
MTT: 3-4,5-dimethylthiazol-2-yl2,5-diphenyl-2H-tetrazolium bromide
PD: Parkinson’s disease
PFA: paraformaldehyde
RT: reverse transcriptase
RT-qPCR: real-time quantitative reverse-transcription polymerase chain reaction
SARS-CoV-2: severe acute respiratory syndrome coronavirus-2
TCID_50_: 50% tissue culture infectious dose
TMPRSS2: transmembrane protease, serine 2
TX-100: Triton X-100
