# Supplementary figures and images for: SARS-CoV-2 infects neurons, astrocytes, choroid plexus epithelial cells and pericytes of the human central nervous system in vitro
Source: J Gen Virol. 2024 Jul 12;105(7):002009. doi: 10.1099/jgv.0.002009 (PMC11317966; doi:10.1099/jgv.0.002009)

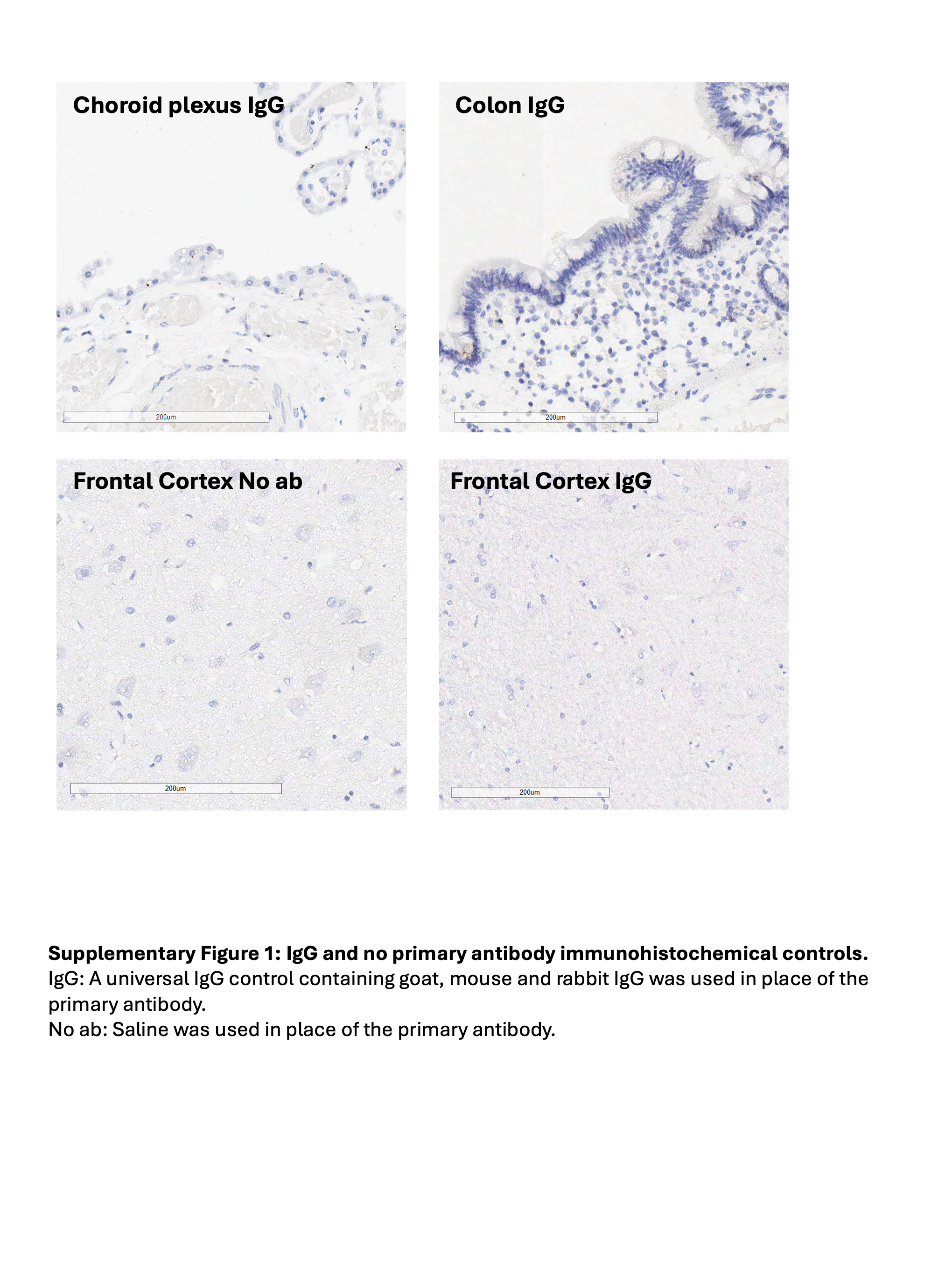

Supplement: Uncited Fig. S1. [file jgv-105-02009-s001.tiff]
